# Amygdala subnuclei volumes, functional connectivity, and social–emotional outcomes in children born very preterm

**DOI:** 10.1093/texcom/tgac028

**Published:** 2022-07-22

**Authors:** Megan Mueller, Benjamin Thompson, Tanya Poppe, Jane Alsweiler, Greg Gamble, Yannan Jiang, Myra Leung, Anna C Tottman, Trecia Wouldes, Jane E Harding, Emma G Duerden, PIANO Study Group

**Affiliations:** Applied Psychology, Faculty of Education, Western University, London N6G 1G7, Canada; School of Optometry and Vision Science, University of Waterloo, Waterloo, Canada; Centre for Eye and Vision Research, 17W Science Park, Hong Kong; Liggins Institute, University of Auckland, Auckland, New Zealand; Liggins Institute, University of Auckland, Auckland, New Zealand; Centre for the Developing Brain, King’s College London, London, UK; Department of Paediatrics: Child and Youth Health, University of Auckland, Auckland, New Zealand; Liggins Institute, University of Auckland, Auckland, New Zealand; Liggins Institute, University of Auckland, Auckland, New Zealand; Department of Paediatrics: Child and Youth Health, University of Auckland, Auckland, New Zealand; Discipline of Optometry and Vision Science, University of Canberra, Canberra, Australia; Liggins Institute, University of Auckland, Auckland, New Zealand; Neonatal Services, Royal Women’s Hospital, Melbourne, Australia; Department of Psychological Medicine, University of Auckland, Auckland, New Zealand; Liggins Institute, University of Auckland, Auckland, New Zealand; Applied Psychology, Faculty of Education, Western University, London N6G 1G7, Canada

**Keywords:** amygdala subnuclei, functional connectivity, social–emotional development

## Abstract

Children born very preterm can demonstrate social-cognitive impairments, which may result from limbic system dysfunction. Altered development of the subnuclei of the amygdala, stress-sensitive regions involved in emotional processing, may be key predictors of social-skill development. In a prospective cohort study, 7-year-old children born very preterm underwent neurodevelopmental testing and brain MRI. The Child Behavioral Checklist was used to assess social–emotional outcomes. Subnuclei volumes were extracted automatically from structural scans (*n* = 69) and functional connectivity (*n* = 66) was examined. General Linear Models were employed to examine the relationships between amygdala subnuclei volumes and functional connectivity values and social–emotional outcomes. Sex was a significant predictor of all social–emotional outcomes (*P* < 0.05), with boys having poorer social–emotional outcomes. Smaller right basal nuclei volumes (*B* = -0.043, *P* = 0.014), smaller right cortical volumes (*B* = -0.242, *P* = 0.02) and larger right central nuclei volumes (*B* = 0.85, *P* = 0.049) were associated with increased social problems. Decreased connectivity strength between thalamic and amygdala networks and smaller right basal volumes were significant predictors of greater social problems (both, *P* < 0.05), effects which were stronger in girls (*P* = 0.025). Dysregulated maturation of the amygdala subnuclei, along with altered connectivity strength in stress-sensitive regions, may reflect stress-induced dysfunction and can be predictive of social–emotional outcomes.

## Introduction

Preterm birth (<37 weeks’ gestation) is highly prevalent, occurring in 12–13% of deliveries, and is increasing worldwide (Goldenberg et al. 2008; Vogel et al. 2018). Preterm birth accounts for the majority of perinatal mortality as well as a significant proportion of long-term morbidity in infants. Developmental delays in terms of motor, cognitive, and language outcomes have been reported in the preterm population, with those born at earlier gestations at most risk (Wen et al. 2004; Løhaugen et al. 2010; Figueras et al. 2011; Goyen et al. 2011; Rathbone et al. 2011; Moore et al. 2012; Nosarti et al. 2012; Arpi and Ferrari 2013; Lax et al. 2013; Potharst et al. 2013; Serenius et al. 2013).

Social–emotional difficulties are also frequently reported in children born very preterm (<32 weeks’ gestation) and remain a key area of concern for parents (Arpi and Ferrari 2013) (Ritchie et al. 2015; Whitley et al. 2016; Mathewson et al. 2017; Hosozawa et al. 2021). A recent large-scale meta-analysis of adults born preterm indicated that early birth was associated with fewer romantic partnerships and that adults born preterm were less likely to have children. These associations were stronger for those born at earlier gestations (Mendonça et al. 2019). Generally, social–emotional difficulties include challenges interacting and communicating within a social context, as well as challenges with regulating emotions (Happé and Frith 2014). In very preterm-born children, these social challenges can even be evident in the first and second years after birth (Landry et al. 1990; Spittle et al. 2009), suggestive of an early brain adaptation.

Social–emotional challenges in preterm-born infants and children are associated with delayed brain development (Rogers et al. 2012; Cismaru et al. 2016; Cheong et al. 2017; Dean et al. 2021; Gilchrist et al. 2021), particularly in limbic regions. Social–emotional challenges in preterm-born individuals may result from enhanced vulnerability in the amygdala (Scheinost et al. 2016; Johns et al. 2019), a key element of the limbic system involved in emotional processing (LeDoux 2003; LeDoux 2012; Rolls 2015). The amygdala demonstrates exponential growth until school age (Giedd et al. 1996; Schumann et al. 2004; Ostby et al. 2009; Uematsu et al. 2012) and may be particularly sensitive to the early exposure to the extrauterine environment resulting from preterm birth. Longitudinal and cross-sectional studies of infants and children born preterm have reported alterations in the development of the whole amygdala (Cismaru et al. 2016; Chau et al. 2019; Johns et al. 2019), which may underlie difficulties with processing affect (Potharst et al. 2013) and social–emotional functioning.

Recent advances in atlas-based MRI-segmentation methods now permit the study of the macrostructure of the amygdala subnuclei (Saygin et al. 2017). The lateral, basal, and accessory-basal nuclei make up the basolateral complex (BLA), which is the main input nucleus to the amygdala (LeDoux et al. 1990; LeDoux 2007; Shah et al. 2019). These nuclei receive sensory information from the hippocampus and prefrontal cortices and send projections to the orbital frontal cortex (OFC), dorsal and ventral striatum, the anterior cingulate, and insular cortices (Davis and Whalen 2001; Cardinal et al. 2002). The central and medial nuclei are output subnuclei of the amygdala. The output nuclei mediate aspects of fear and anxiety processing through the projections to the hypothalamus, basal forebrain, and brainstem sites, which are involved in different aspects of stress responses (Nordahl et al. 2012). Atypical maturation of the input and output nuclei and their projections to limbic cortices and brain regions involved in the stress response system (Sinha et al. 2015) may underlie social emotional impairments. Both structural and/or functional alternations may underlie the social–emotional difficulties seen in children born preterm (Forbes and Dahl 2010; Papini et al. 2016; Urbain et al. 2019). Therefore, an understanding of structural and functional differences, as well as the interaction between the two, is critical in the potential early identification of social–emotional difficulties in this vulnerable group of children.

Early difficulties with socio-emotional functioning may place children at risk for later behavioral and psychiatric problems (Briggs-Gowan and Carter 2008; Treyvaud et al. 2012). Furthermore, research has suggested that social functioning plays a clinically relevant role in predicting the course and outcomes of several psychiatric disorders (Cannon et al. 1997; van Os et al. 2010). To make accurate predictions of risk and to guide and implement support strategies, further studies are needed to increase our understanding of the mechanisms underlying the specific socioemotional concerns of individuals who were born preterm or with low birth weight.

In a longitudinal cohort of very preterm born (<30 weeks’ gestation) school-aged children followed since birth, we aimed to determine the association among social–emotional outcomes and amygdala subnuclei volumes. Secondly, we aimed to examine the relationships between functional connectivity strength and social–emotional outcomes. Our third aim was to ascertain how the function–structure relationships in the limbic system predict social–emotional outcomes at school age in children born very preterm.

## Methods

### Patients

Neonates born very preterm (24–30 weeks’ gestation) who were admitted to the neonatal intensive care unit (NICU) at National Women’s Hospital, Auckland, New Zealand were enrolled in the study over a three-year period (2005–2008). Neonates were excluded from the study based on the following criteria: congenital malformation or syndrome, or ultrasound evidence of a large parenchymal hemorrhagic infarction.

### Design

Infants were enrolled in the PIANO study at 7 years of age, as described previously (Alsweiler et al. 2012; Tottman et al. 2020). Informed consent was obtained from a parent or legal guardian. The study was approved by the Northern B ethics committee (NTY/12/05/035) and the Auckland District Health Board (ADHB 5486).

### Neuropsychological testing

Parents completed the Child Youth and Behavioral Checklist (CBCL) (CAM, 2019). This is a component of the Achenbach System of Empirically-based Assessment (ASEBA), which is used to detect emotional and behavioral challenges in children (CAM, 2019). The CBCL has sound internal reliability with Cronbach’s alpha scores ranging from 0.71 to.89 for each syndrome scale (Nakamura et al. 2009). The CBCL scores were assessed for the Social, Activities and School Competency, Social Problems and Aggression subscales CBCL subscale score categories are Normal (<93rd percentile), Borderline (93rd–98th percentile) and Clinical (>98th percentile).

### Magnetic resonance imaging

Children underwent anatomical and functional MRI on a 3 T scanner (Siemens, Skyra, Erlangen, Germany). Anatomical images were acquired using an MPRAGE pulse sequence ([repetition time] TR, 2000 ms, [echo time] TE, 3510 ms, [inversion time] TI, 1010 ms, slice thickness, 0.85 mm, FOV, 210 × 210 mm). Resting-state fMRI was acquired using an echo-planar imaging sequence (TR, 3200 ms, TE, 40ms, FOV, 147 × 147mm, matrix, 64 × 64, slice thickness, 3 mm).

### Subnuclei segmentation

The total cerebral volume, whole amygdala and nine of the amygdala subnuclei were segmented automatically using FreeSurfer (https://surfer.nmr.mgh.harvard.edu/) version 6.0. The algorithm is based on Bayesian inference, and the amygdala atlas was developed using *ex vivo* human sections of the medial temporal lobes (*n* = 10).

In the current study, the amygdala was segmented into the central (Ce), lateral, basal, accessory basal, cortical, medial, and paralaminar nuclei as well as the corticoamygdaloid transition area (CTA) and the anterior amygdaloid area (AAA). The volumes of the nuclei forming the BLA complex (basal, lateral, and accessory basal nuclei) were summed and were used as predictor variables in the subsequent analyses.

Visual inspection was used for quality control of the postprocessed amygdala segmentations using the Freeview image viewing platform within the FreeSurfer package. Postprocessed images were visually inspected to determine the segmentation accuracy using anatomical atlases as reference material (Amunts et al. 2005; Solano-Castiella et al. 2010). Subnuclei volumes were automatically extracted using custom software available on the Developing Brain Lab GitHub site: (https://github.com/DevelopingBrainLab/amygdala_segmentation_Freesurfer).

### Functional connectivity

Data from the resting-state fMRI were preprocessed using the FMRIB Software Library (FSL; version 5.0.6). Slice timing and motion correction, spatial smoothing, band-pass filtering (suppressing physiological noise), and whole-brain tissue extraction were included in the pre-processing. Data were nonlinearly registered to a standardized MRI template in Montreal Neurological Institute (MNI) space. Functional connectivity was examined using a group temporal concatenation independent components analysis (ICA), available in FSL using Multivariate Exploratory Linear Decomposition into Independent Components (MELODIC, version 4.0). This produced a set of independent components common to the whole group. All resting-state components were visually inspected. Time courses were extracted from the amygdala, cingulate, and thalamic networks, and the correlation coefficients were calculated.

### Statistical analyses

All statistical analyses were carried out using SPSS (version 26, Statistical Package for the Social Sciences, IBM, Chicago, IL). To address our first aim, general linear models (GLM) were used to examine the association of amygdala subnuclei volumes with social–emotional outcomes on the CBCL, adjusting for gestational age (GA) at birth, birth weight *z* score (Thompson et al. 1994), age at scan, cerebral volumes, and biological sex. Birth weight *z* score reflects growth before birth and is expressed in standard deviations from the mean for biological sex and GA that are independent of absolute weight. As part of our plan to examine laterality differences, the analyses were completed separately for the left and right subnuclei volumes. As we had one a priori hypothesis regarding the association of social–emotional challenges and amygdala subnuclei volumes, the alpha level for the statistical test was set at 0.05.

To address aim two, the time courses from the thalamus, amygdala and cerebral cortices were extracted. Correlation coefficients (Pearson's R) describing the connectivity strength between networks were calculated for each participant. To assess the relationship between social–emotional outcomes at 7 years of age and functional connectivity strength between the subcortical and cortical resting-state networks, we used a GLM, with the correlation coefficients describing thalamocortical connectivity strength entered as the independent variables and the social–emotional outcomes as dependent variables. Analyses were adjusted for GA at birth, birth weight *z* score, age at scan and biological sex. We had one hypothesis regarding the association between the functional connectivity strength and the social–emotional outcomes, and the alpha level was set at 0.05.

To address our third aim, GLMs were used to examine the interaction between functional connectivity strength, subnuclei volumes, and biological sex in predicting social–emotional outcomes at 7 years of age. Analyses were adjusted for GA at birth, birth weight *z* score, age at scan, total cerebral volumes, and biological sex. We had two hypotheses regarding the association between the functional connectivity strength and the amygdala and limbic areas, and the alpha level was set at 0.03, using the Bonferroni correction method.

## Results

Participants included 113 7-year-old preterm born children (boys = 56, girls = 57, mean age = 7.5 years). The mean GA of the infants at birth was 25.6 weeks, and the average birthweight was 931 g. Based on the quality of the MRI scans, amygdala subnuclei volumes were extracted for 69 (boys = 33, girls = 36) participants, and functional connectivity was assessed in 66 participants (boys = 33, girls = 33).

### Neuropsychological outcomes

Parents of 113 children completed the CBCL. The majority of total competence scores fell into the normal range (*n* = 76[38 male]), with some falling in the clinical range (*n* = 28 [16 male]) and the borderline range (*n* = 8[4 male]). The scores for the three competency scales and the five syndrome scales are provided in Table 1.

**Table 1:** Children’s behavior checklist: Competency scale scores and syndrome scale scores for boys and girls.

Competency/substantial scale	Boys	Girls	*P-*value
Social scale (SE)	6.56 (.770)	7.42 (.7930)	.040^a^
School scale (SE)	4.37 (.429)	4.61 (.4412)	.312
Activities scale (SE)	9.50 (.669)	9.86 (.6871)	.315
Social problems (SE)	4.07 (1.039)	2.97 (1.068)	.049^a^
Aggressive behavior (SE)	5.34 (.648)	3.64 (.666)	.068
Withdrawn/depressed (SE)	1.84 (.267)	1.91 (.274)	.844
Rule Breaking behavior (SE)	2.15 (.270)	1.51 (.278)	.100
Thought problems (SE)	2.65 (.381)	2.00 (.391)	.237

Values represent the estimated marginal means of the syndrome scale scores, adjusting for GA at birth, birth weight z score, age at scan and cerebral volumes. CBCL, Children’s Behavior Checklist, SE, standard error.

^a^Statistically significant, *P* < 0.05.

### Subnuclei volumes

**Fig. 1:**
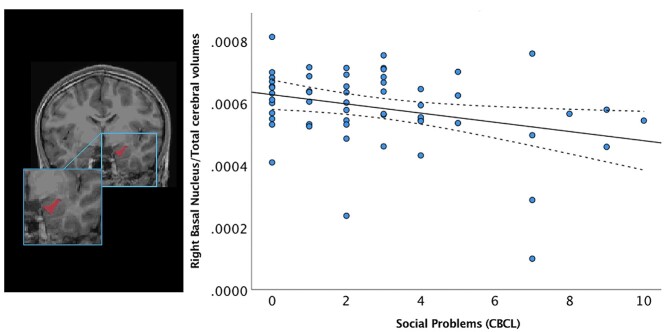
Children born preterm with greater social problems scores assessed with the CBCL had smaller right basal volumes (*B* = −0.043, *P* = 0.014). Dashed lines represent 95% confidence intervals.

To address our first aim, the association of amygdala subnuclei volumes with social–emotional outcomes on the CBCL was assessed using a GLM, adjusting for GA at birth, birth weight *z* score, age at scan, cerebral volumes, and biological sex. Smaller right basal nuclei volumes predicted greater social problems (*B* = −0.043, *P* = 0.014) (Fig. 1) and aggressive behaviors scores *(B* = −0.14, *P* = 0.012). Smaller right cortical nuclei volumes were also associated with higher scores on the social problems scale (*B* = −0.242, *P* = 0.020). Additionally, smaller right medial nuclei volumes predicted higher scores on the aggressive behavior scale (*B* = −0.07, *P* = 0.033). However, larger right central nuclei volumes predicted higher scores on the social problems scale *(B* = 0.85, *P* = 0.049). Biological sex was a significant predictor of the outcomes in all models (*P* < 0.05).

We subsequently assessed the interactions among the subnuclei volumes and biological sex. Significant interactions were identified between biological sex and the right basal nuclei volumes in predicting social problems (*P* = 0.002). Both boys (*B* = −0.007, *P* = 0.012) and girls (*B* = −0.009, *P* = 0.002) with smaller right basal nuclei volumes had higher social problems scores, with the association being stronger in girls. Significant interactions were identified between biological sex and the right cortical nuclei in predicting social problems (*P* = 0.005) such that both boys (*B* = −0.112, *P* = 0.003) and girls (*B* = −0.083, *P* = 0.031) with smaller right cortical nuclei volumes had higher social problems scores, with the association being stronger in boys. When examining sex differences, only girls showed a significant negative association between right basal nuclei volumes and higher aggressive behaviors scores (*B* = −0.011, *P* = 0.043). There were no significant interactions between right central and medial nuclei volumes and sex in predicting social–emotional outcomes. No significant associations were found among left subnuclei volumes and social–emotional outcomes.

### Connectivity strength

**Fig. 2:**
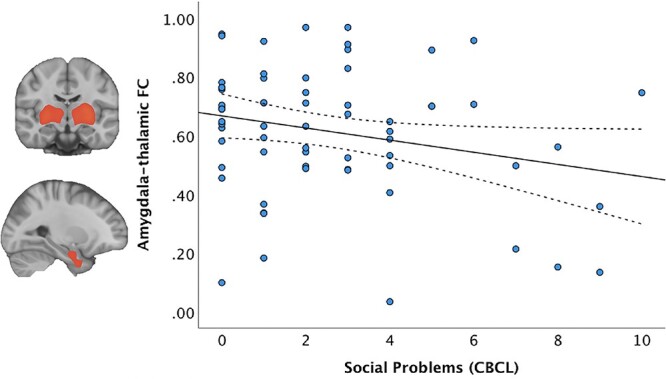
FC between the amygdalar and thalamic networks in preterm born children and the association with social problems scores assessed with the CBCL. Preterm born children with increased social problems had reduced connectivity strength between the amygdala-thalamic networks (*B* = −8.74, *P* = 0.022). Dashed lines represent 95% confidence intervals.

Decreased connectivity strength between the thalamic and amygdala networks significantly predicted higher Social Problems scores (*B* = −8.74, *P* = 0.022) (Fig. 2), when adjusting for GA at birth, birth weight z score, age at scan, and biological sex.

### Functional and structural relationship

Connectivity strength between the thalamic and amygdala resting state networks was found to interact with right basal nuclei volumes and biological sex in predicting social problems scores (*P* = 0.019). The significant interaction between resting state networks and right basal nuclei was predictive of social problem scores in girls (*P* = 0.025), but not in boys.

## Discussion

In this cohort of school-aged children born very preterm, structural alterations in the amygdala and its connectivity predicted social–emotional challenges. More specifically, children with smaller right basal, medial, and cortical nuclei volumes had increased social–emotional difficulties, whereas larger right central nuclei volumes were associated with increased social–emotional difficulties. Additionally, reduced connectivity strength between amygdalar and thalamic networks was associated with poorer social outcomes. Correlations between functional and structural differences were related to social difficulty in girls, but not boys born preterm.

Previous research indicates that preterm born children display atypical development of the amygdala (Cismaru et al. 2016; Chau et al. 2019; Johns et al. 2019), and this may be related to difficulties processing affect in others. The current study identifies specific regions in which these alterations may occur and related these two areas of social difficulty. More specifically, the volumes of structures in the basolateral complex, the main input nuclei (LeDoux et al. 1990; LeDoux 2007; Shah et al. 2019), were correlated with social–emotional challenges. The finding that children with smaller right basal nuclei had greater difficulty socially and a higher prevalence of aggressive behaviors is supported by previous research (Rogers et al. 2012; Cismaru et al. 2016; Cheong et al. 2017; Dean et al. 2021; Gilchrist et al. 2021), which suggests that delayed brain development, particularly in limbic regions, is associated with social–emotional challenges. Interestingly, the same pattern is not seen in relation to childhood anxiety in preterm born children (Qin et al. 2014), suggesting that the correlation between reduced volumes in the amygdala and school age outcomes is observed solely in relation to later externalizing behaviors. Sinha et al. (2015) identified the critical role of the input and output subnuclei of the amygdala in the stress response system, which is highly sensitive to social challenges in children (Flinn 2006). The current findings support this relation between input and output nuclei and social challenges, such that the volumes of the main output nuclei (central and medial nuclei) were correlated with social functioning.

Neural pathways between the thalamus to the amygdala are particularly important in emotional learning. Reduced functional connectivity strength between the amygdalar and thalamic resting state networks is common in children born very preterm (Scheinost et al. 2016); however, the relationship to social–emotional functioning has been unclear (Johns et al. 2019). The current findings suggest reduced functional connectivity between thalamic and amygdalar networks at age 7 is predictive of increased social challenges in preterm born children. This finding is supported by previous research (Johns et al. 2019) reporting that altered amygdala connectivity is correlated with social impairment in adults born preterm. However, other research (Kanel et al. 2022) has reported no association between amygdala resting state connectivity at birth and social function between the ages of 4 and 7. Therefore, the current findings, along with those reported elsewhere (Johns et al. 2019, Kanel et al. 2022), suggest that perhaps this association develops later in childhood, potentially reflecting a long-term impact of early life stress on neurodevelopment (Smith et al. 2011; Grunau 2013).

Anatomical distribution of the brain regions involved in social–emotional processing is complex, resulting in the need for a clear understanding of both structural and functional differences for potential early identification of social–emotional challenges. In girls, functional connectivity strength between amygdalar and thalamic networks, in addition to right basal nuclei volumes was predictive of social challenges. This is congruent with other studies reporting that both structural and functional alterations in the frontoparietal areas are associated with poorer social–emotional functioning (Urbain et al. 2019). The current findings provide support for sex differences in the associations among brain structure-relationships and social outcomes in a young group of children. Previous research on sex differences on structure–function relationships and behavior suggests that perhaps these differences are maladaptive (Grabowska 2017), which may explain the differences observed in the current data.

While this study has the strengths of a large sample size, both structural and functional analysis and the ability to control for demographic variables, some limitations have also been identified. The current study did not have a control group of children born at term for comparison. Additionally, the social–emotional outcomes were only assessed through parent report.

Future studies should investigate the trajectory of structural and functional neurological differences throughout development in children born very preterm. Identification of the precise time period where these anatomical changes can be detected will allow for the early identification of potential social–emotional challenges in this population. Additionally, future research should evaluate the role of interventions in improving outcomes for preterm born children who have atypical brain structure and connectivity patterns.
